# Supplementary figures and images for: Laminar specificity of the auditory perceptual awareness negativity: A biophysical modeling study
Source: PLoS Comput Biol. 2023 Jun 29;19(6):e1011003. doi: 10.1371/journal.pcbi.1011003 (PMC10337981; doi:10.1371/journal.pcbi.1011003)

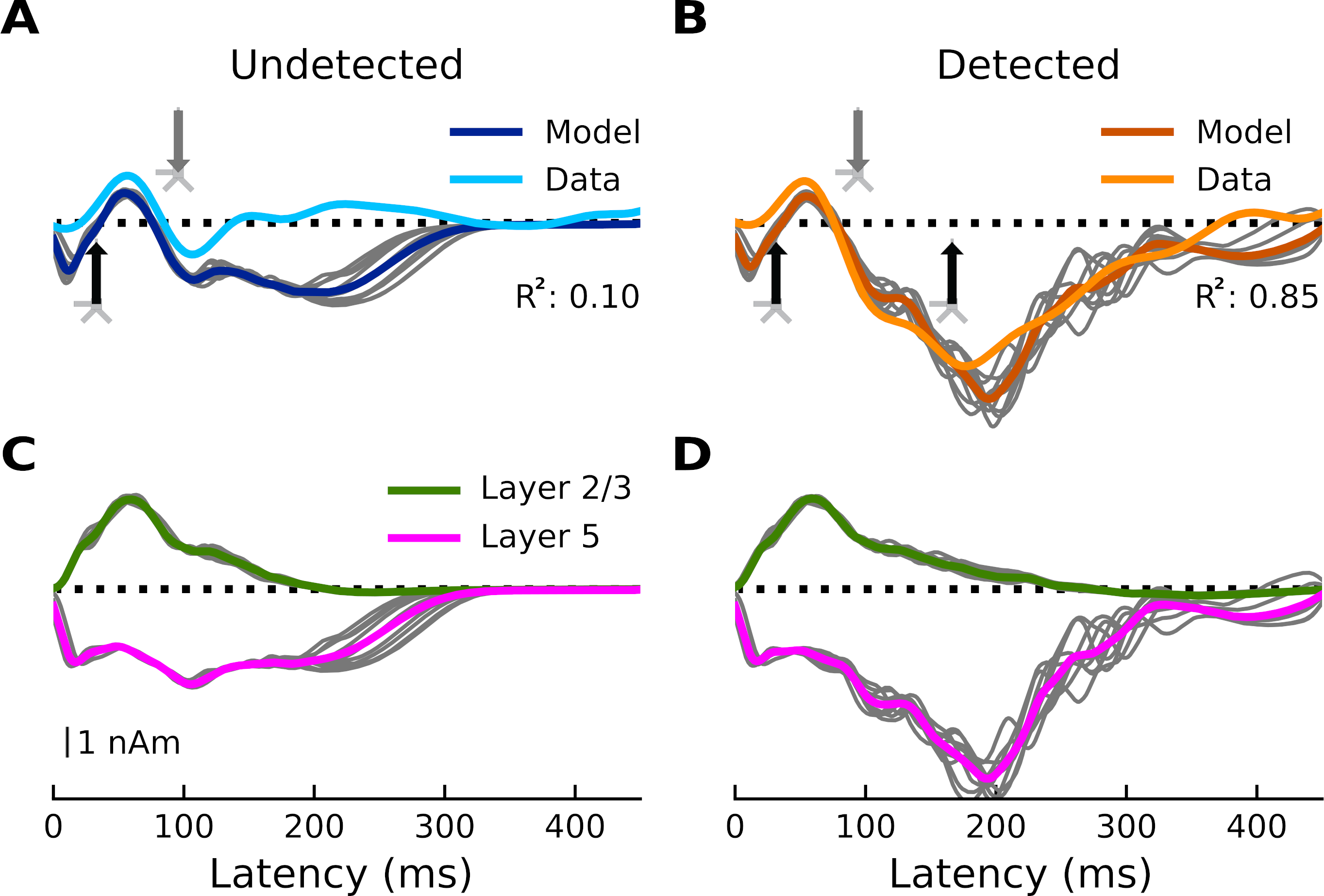

Supplement: S1 Fig — (A) Model output (dark blue) and data (light blue) for undetected target tones. A proximal input (36 ms) followed by a distal input (84.3 ms) drive the network. R2 between empirical and simulated data is 0.10, RMSE is 1.41 nAm. (B) Model output (dark orange) and data (light orange) for detected target tones. The same proximal and distal inputs used to model the response to undetected target tones, and an additional proximal input (169.3 ms), drive the network. R2 between empirical and simulated data is 0.85, RMSE is 0.80 nAm. (C-D) Laminar profiles for the responses to undetected and detected target tones. The corresponding input parameter values are displayed in S4 Fig. The network from which the simulated dipole activity arises consists of 60,000 cells. (TIF) [file pcbi.1011003.s001.tif]

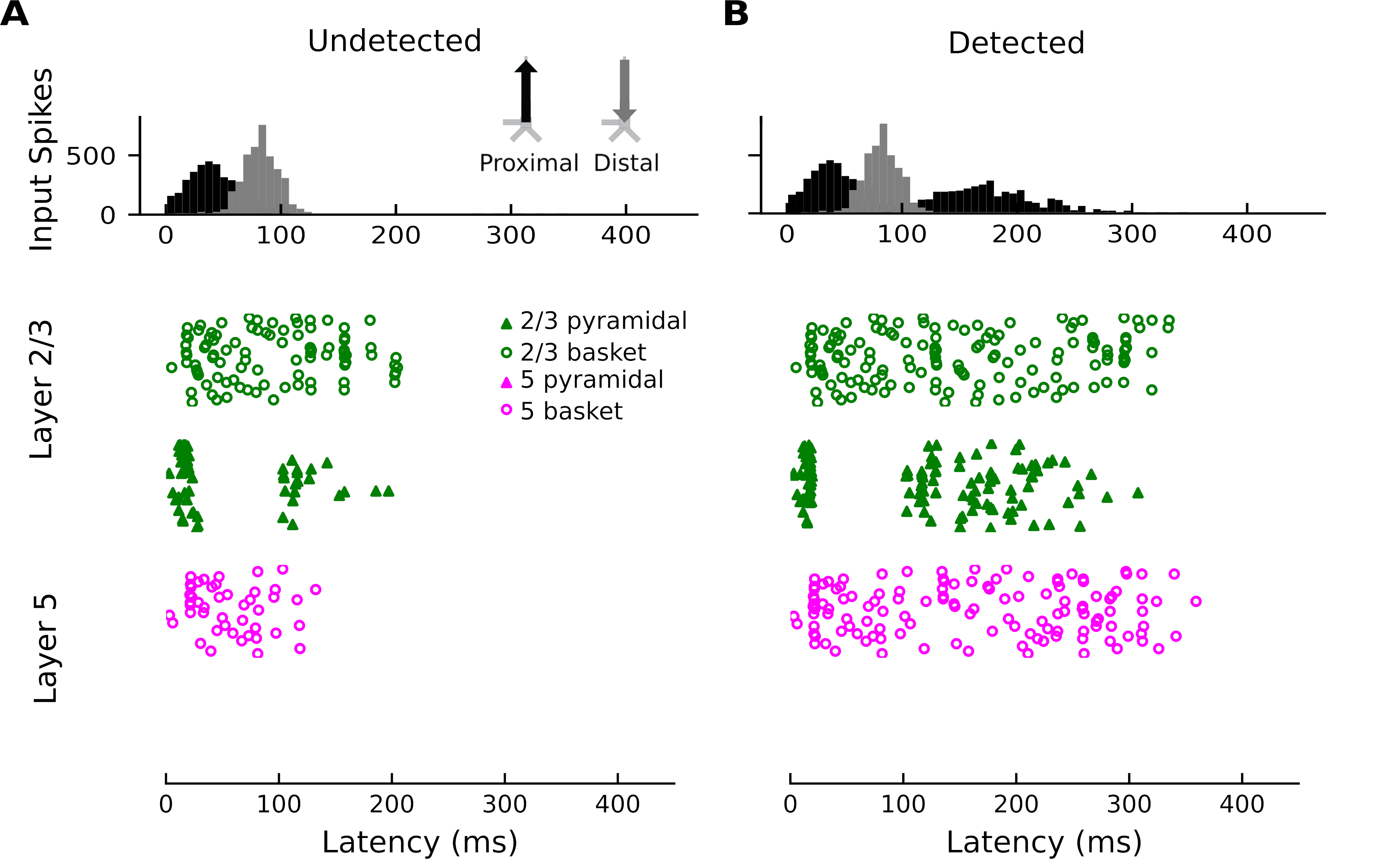

Supplement: S2 Fig — (A) Network spiking activity for undetected target tones. (B) Network spiking activity for detected target tones. Note in both cases the complete absence of spiking activity in L5 pyramidal neurons. (TIF) [file pcbi.1011003.s002.tif]

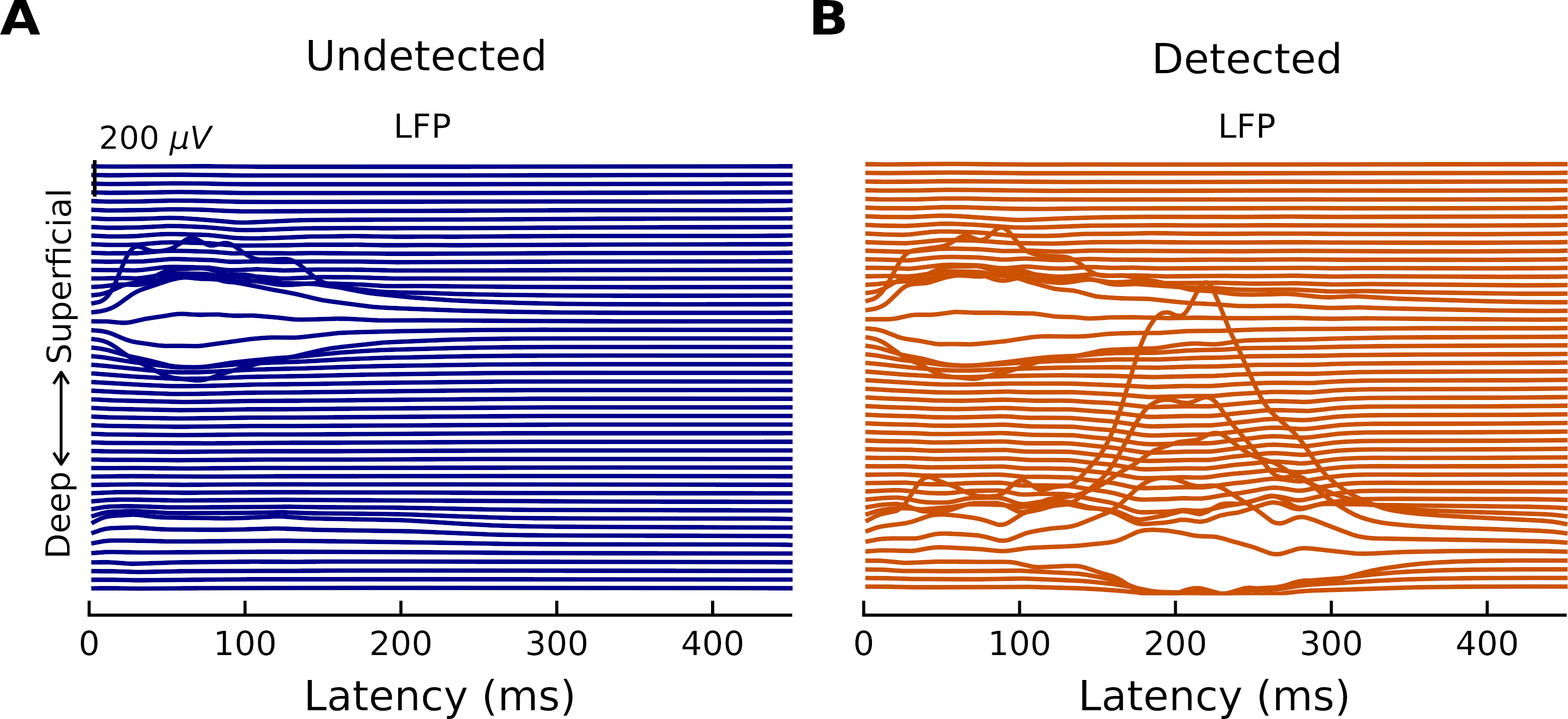

Supplement: S3 Fig — (A) Simulated LFP to undetected target tones. (B) Simulated LFP to detected target tones. (TIF) [file pcbi.1011003.s003.tif]

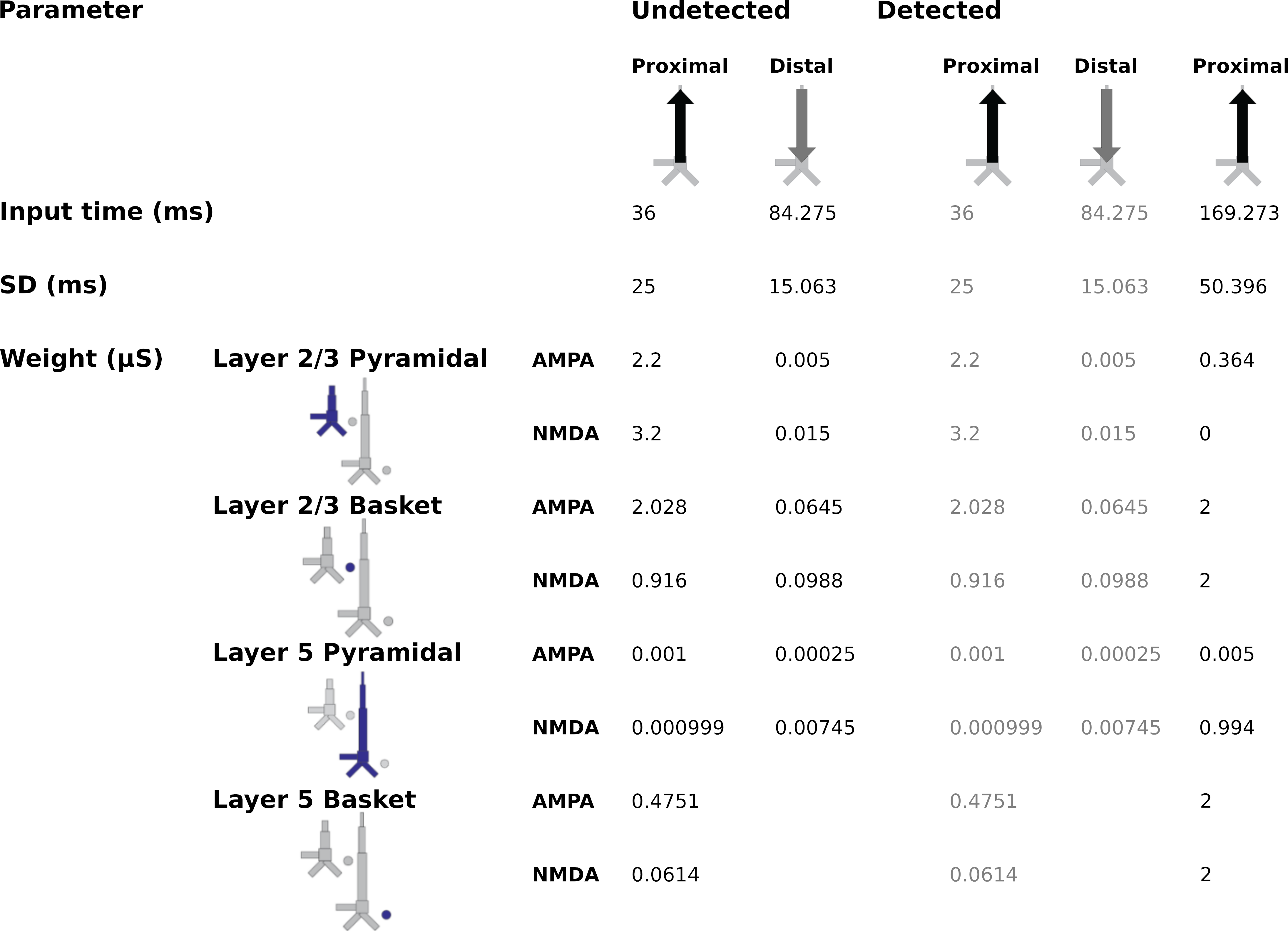

Supplement: S4 Fig — The canonical circuit model was modified by increasing the conductance of the GABAB receptors on the perisomatic compartment of L5 pyramidal neurons. (TIF) [file pcbi.1011003.s004.tif]

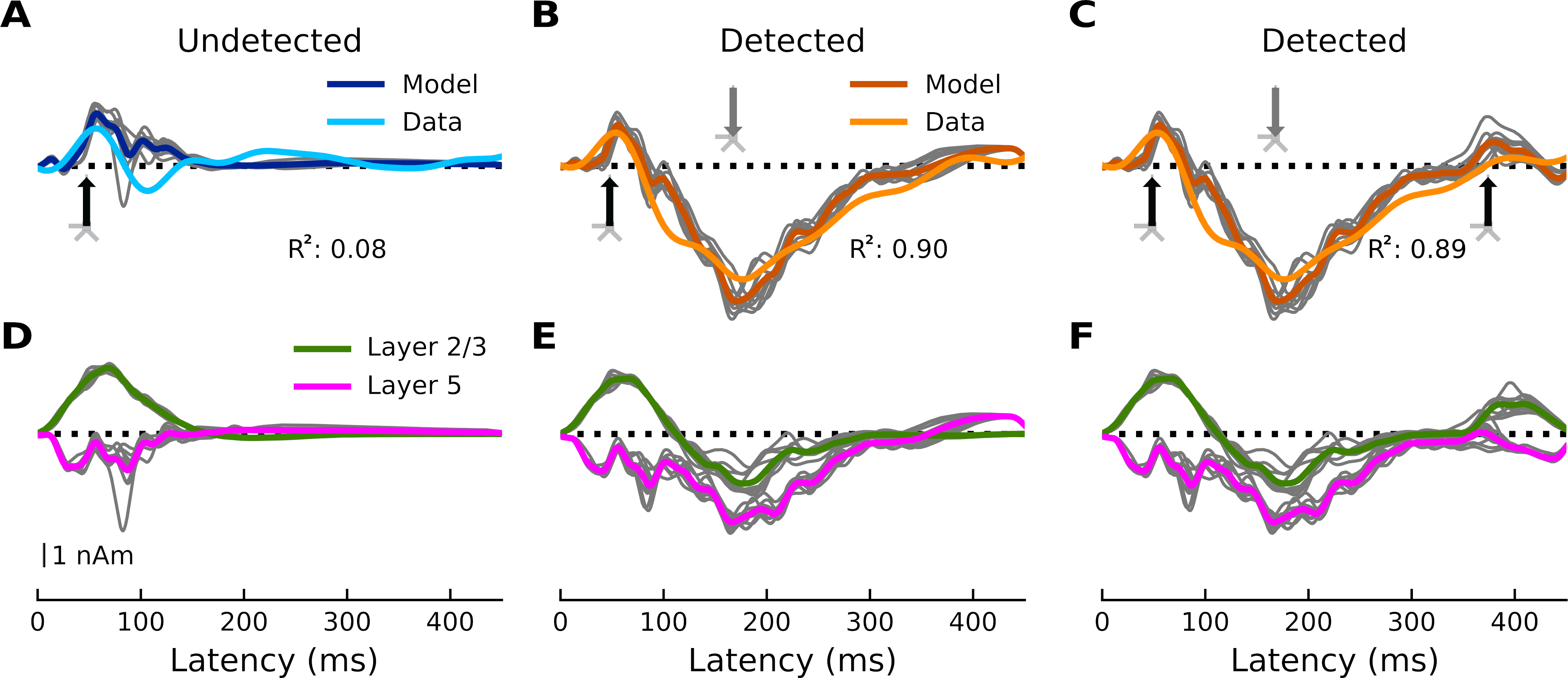

Supplement: S5 Fig — (A) Model output (dark blue) and data (light blue) for undetected target tones. A single proximal input (47.8 ms) drives the network. R2 between empirical and simulated data is 0.08, RMSE is 0.61 nAm. (B) Model output (dark orange) and data (light orange) for detected target tones for the reduced input sequence model. The same proximal input that was used to model the response to undetected target tones, and a distal input (154.0 ms) drive the network. R2 between empirical and simulated data is 0.90, RMSE is 0.70 nAm. (C) Model output (dark orange) and data (light orange) for detected target tones for the proximal-distal-proximal model. The same proximal input that was used to model the response to undetected target tones, a distal input (154.0 ms), and an additional proximal input (395.4 ms) drive the network. R2 between empirical and simulated data is 0.89, RMSE is 0.73 nAm. (D-F) Laminar profiles for the responses to the undetected and detected target tones. The corresponding input parameter values are displayed in S8 and S9 Figs. The network from which the simulated dipole activity arises consists of 60,000 cells. (TIF) [file pcbi.1011003.s005.tif]

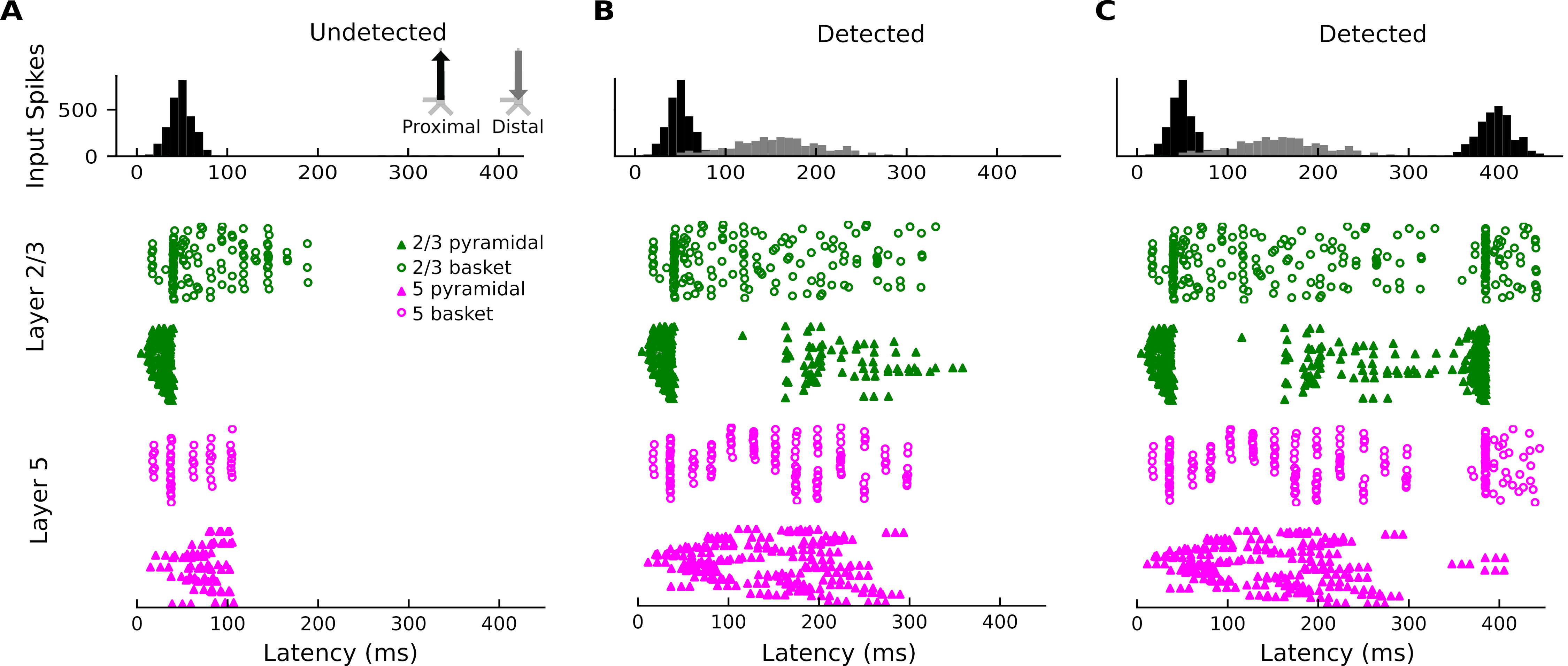

Supplement: S6 Fig — (A) Network spiking activity for undetected target tones. (B) Network spiking activity for detected target tones for the reduced input sequence. (C) Network spiking activity for the detected target tones for the proximal-distal-proximal input sequence. (TIF) [file pcbi.1011003.s006.tif]

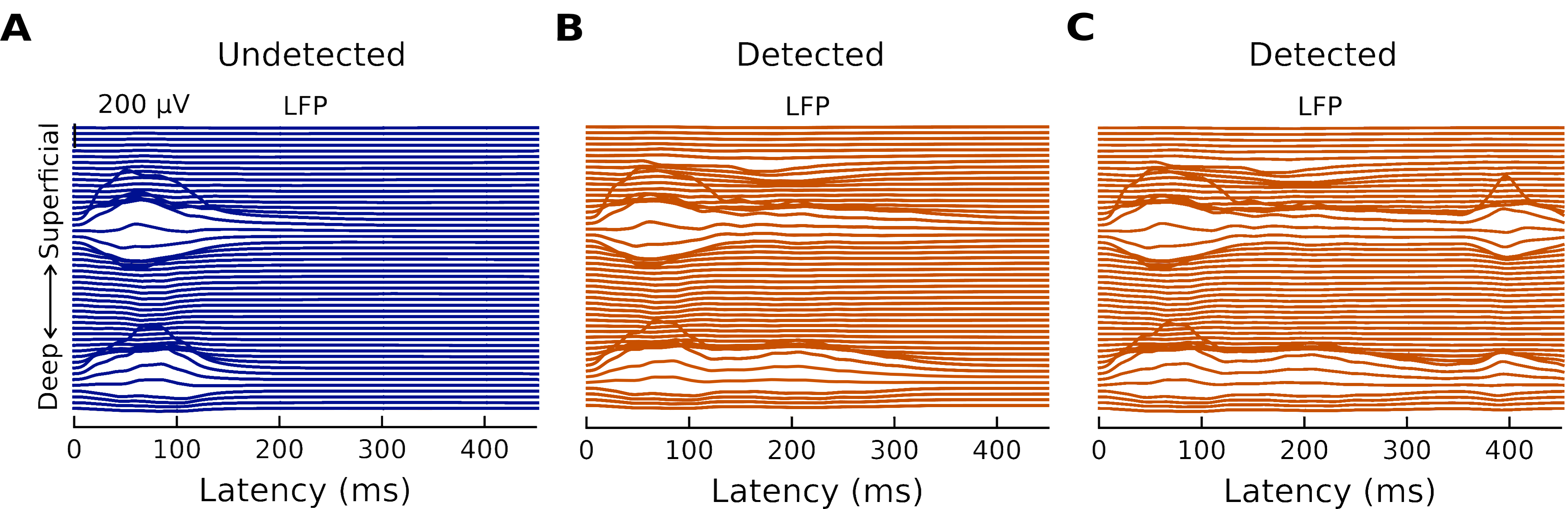

Supplement: S7 Fig — (A) Simulated LFP for undetected target tones. (B) Simulated LFP for detected target tones for the reduced input sequence. (C) Simulated LFP for detected target tones for the proximal-distal-proximal input sequence. (TIF) [file pcbi.1011003.s007.tif]

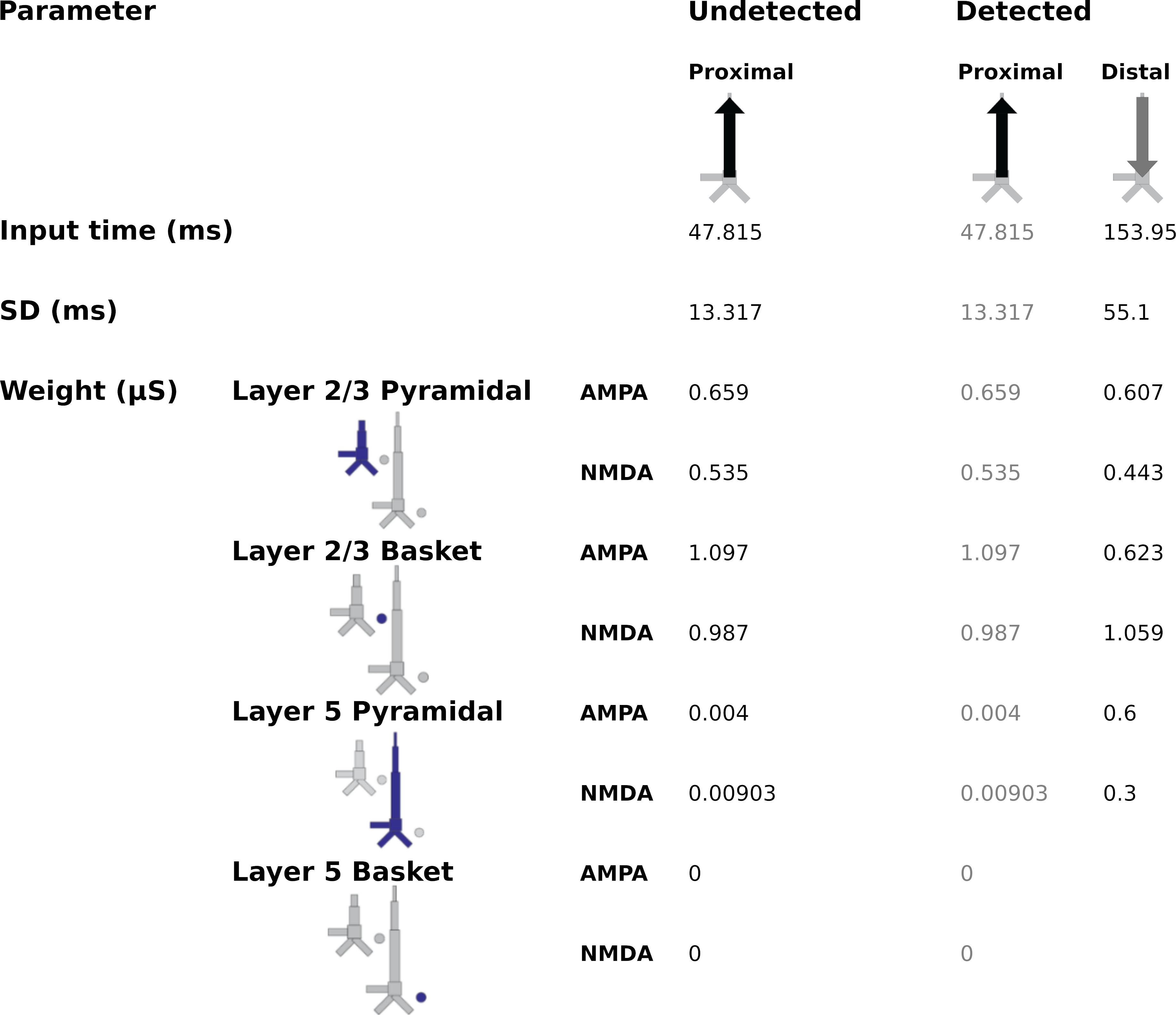

Supplement: S8 Fig — The model column used was the calcium model column that consists of a more biologically accurate distribution of Ca2+ channels on L5 pyramidal neurons compared to HNN’s original Jones 2007 model column. (TIF) [file pcbi.1011003.s008.tif]

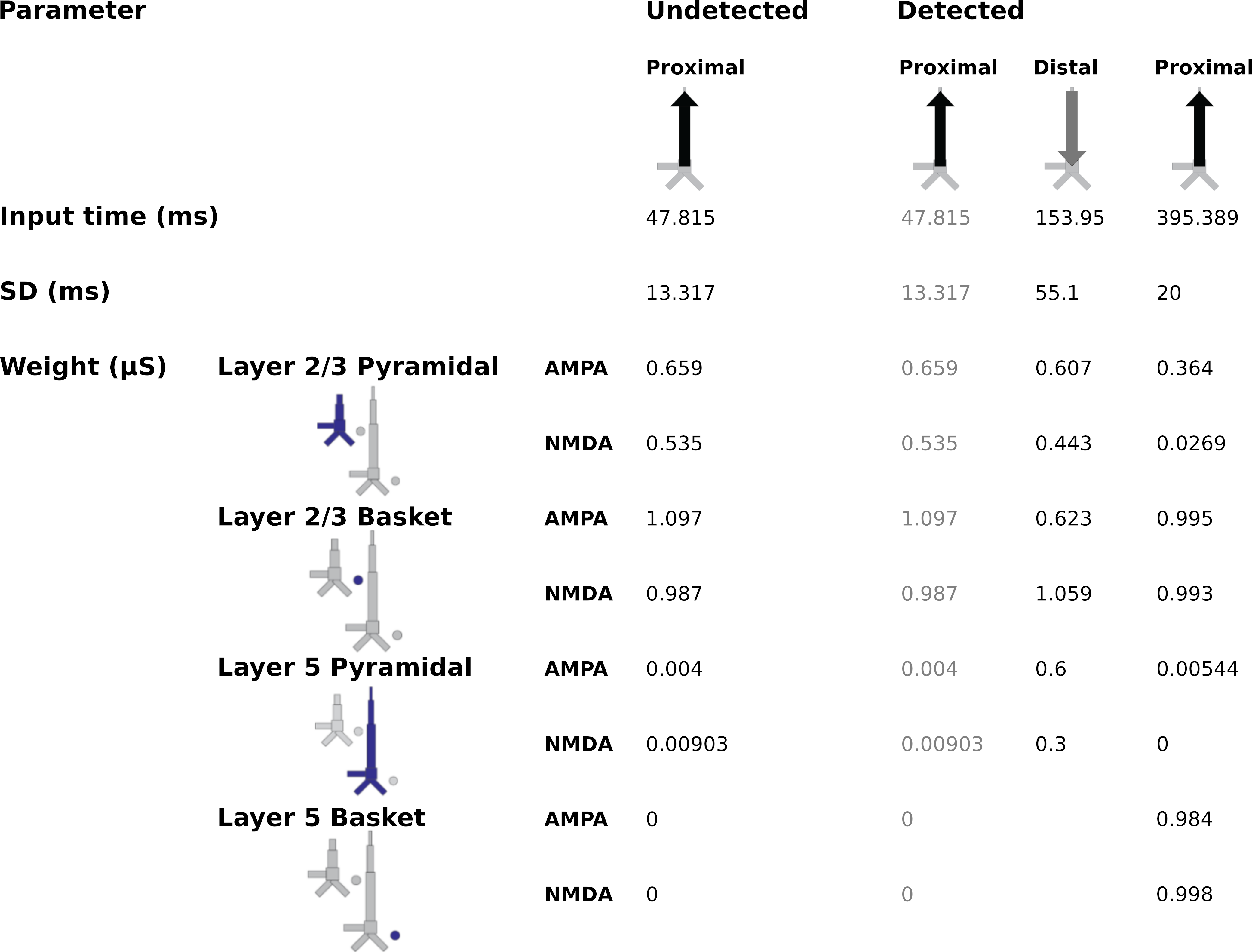

Supplement: S9 Fig — The model column used was the calcium model column that consists of a more biologically accurate distribution of Ca2+ channels on L5 pyramidal neurons compared to HNN’s original Jones 2007 model column. (TIF) [file pcbi.1011003.s009.tif]
