# Supplementary material for: Laminar specificity of the auditory perceptual awareness negativity: A biophysical modeling study
Source: PLoS Comput Biol. 2023 Jun 29;19(6):e1011003. doi: 10.1371/journal.pcbi.1011003 (PMC10337981; doi:10.1371/journal.pcbi.1011003)
Supplement: Attachment — Submitted filename: Response_to_Reviewers.pdf [file pcbi.1011003.s010.pdf]

Dear Carolina and Andrew,
Thank you for submitting your research to Plos Comp. Biol. As you will read the reviewers were mostly enthusiastic about your work but raised some minor issues that you will want to address. I also enjoyed reading your contribution. I suggest also adding a color code (or some other marking such as an arrow) to the spectrogram of figure 1 to shown the location of the target stimulus - probably obvious to you.

Best wishes,
Frederic Theunissen
Thanks for this suggestion. We added an arrow to indicate the target tone stream.

General comments:
We found the suggestions from both reviewers to be quite helpful and thank them for their feedback. We've made several substantial changes, bulleted here. Responses are also given in line below the reviewer comments.

- We created an OSF repository to host the data and code necessary to reproduce the modeling work, which is linked in the revised manuscript.
- We added units to the RMSE values and added R^2 values (from simple linear regression) to the model fits to indicate fraction of explained variance for each model. In so doing, we also corrected an error we discovered during our re-analysis that previously led us to plot unoptimized model fits; optimized fits are now plotted in the revised manuscript. This did not substantially change our conclusions.
- We added a two-paragraph section to the Discussion that attempts to relate our findings from modeling awareness-related negativities in the multitone masking paradigm to other paradigms relevant for the study of auditory conscious perception and paradigms that elicit responses that have similar spatiotemporal profiles.
- We included discussion of both integrated information theory and predictive processing theory in network-level theories of conscious perception.
- We eliminated abbreviations where possible (e.g. PN for pyramidal neurons). This certainly made the paper more readable.

Reviewer #1:
This is a highly valuable and unique contribution to the literature on auditory awareness, and I have no doubt that it should be published. I only have a few minor suggestions below, though a fair bit of new writing could be needed to broaden the Introduction and Discussion.

Currently, the Introduction and Discussion are perhaps too narrowly focused on awareness negativity and particular cortical column level mechanisms. However, I wonder if a broader discussion of other types of brain activity and mechanisms would make for a more engaging and impactful paper by truly connecting different grains of understanding of the neural basis of sensory awareness. For example, there is existing modeling of auditory bistable perception by Rankin/Rinzel and Denham, and more recently by David Little et al. in this journal that include biologically realistic mechanisms. Including these in a broader discussion and if possible seeing if any of the concepts in those studies (e.g., inhibition of

competing neural populations, predictive coding) relate to the ones in your study would add a lot of value in moving towards general principles, rather than just trying to explain individual paradigms like informational masking or stream segregation. For example, your model includes inhibitory inputs not surprisingly given their ubiquity in neural circuitry, and you may react that for informational masking, there are no competing representations like in a bistable paradigm. But I would counter that when listening for a target of a particular frequency, there will always be need to inhibit neighboring neural populations that are selective for stimuli of a different frequency.
Thank you for the valuable suggestion. We've broadened our Discussion to include a new section called "Relation to other paradigms" where we highlight the general principles of our model predictions that may play a role in other phenomena like perceptual bistability. We elected to keep the Introduction focused on the background most relevant to our modeling question. We hope this is a good tradeoff of focus early in the manuscript and broader discussion later.

Likewise, your model includes feedback input, which is a key neural architecture for predictive coding models and could be a key concept to explain the generation of the awareness negativities. It is also a possible way to connect awareness negativities to other large responses of similar latencies such as mismatch negativity and ERAN (evoked in studies of music processing, see studies by S. Koelsch, and his review in Trends in cognitive sciences, 2019). And there are models of mismatch negativity generation (that rely on feedback/predictive coding and NMDA receptors) that might be interesting to discuss and compare to your model.
Under the section mentioned above, we make the case that although we do not explicitly model the effects of inhibitory feedback, it likely impacts perception in the multitone masking paradigm, as in auditory streaming paradigms. We also argue that although other negativities like the MMN, ERAN, N2ac, and processing negativity have superficially similar latencies and scalp distributions, the underlying circuits that give rise to each may differ. For example, layer ⅔ may play an important role in the MMN, while the AAN likely arises from amplificatory mechanisms in layer 5.

I would remove all or most of the abbreviations, e.g., PNs. This will improve readability by quite a bit.
Thank you for this suggestion. We've now edited all relevant text to read "pyramidal neurons" instead of "PN", and have only abbreviated "layer V" to "L5", and similar. We agree that this made the manuscript more readable.

Pg 8, there is a mention of the auditory P0 and P1, but I wonder if instead of P0 you are referring to the Pb response. P0 is very short latency and precedes even the Pa middle-latency response, so well before the P1.
Thanks for picking this up. It was an error on our part and has now been corrected to "Pa" and "P1".

Pg. 14, need references to support the statement that "altered GABAB receptor function is associated with a variety of disorders"
Thanks for picking this up. The reference has now been included.

Pg. 17 “network-level theories of consciousness” Does your modeling relate at all to any of the key concepts in information integration theory? If so, it would be nice to point out how.
Thanks for this suggestion. We’ve now included IIT and predictive processing theory in our discussion of network-level theories of consciousness.

Signed Review,
Joel Snyder

Reviewer #2:
This manuscript describes an interesting exploratory modeling study of the potential biophysical sources of the auditory awareness negativity (AAN). The model is fit to MEG data, and the results indicate that synaptic input to the supragranular layers of auditory cortex (AC) best explains the AAN waveforms. Some alternative alternatives to the primary version of the model are investigated, and while these can capture aspects of the AAN data fairly well, they fail to explain the AAN data as fully as the primary model.

Overall the manuscript is well written, with fairly good English usage. In a number of place contractions are used (e.g., "they’re" on page 5, "doesn’t" on pages 14 & 15, and "didn’t" on page 15). It is preferable to avoid use of contractions in technical writing.
Thank you, we removed all contractions.

The modeling approach is well explained in the most part, and there is fairly good discussion and interpretation of the results. Two areas that could do with improved clarity are:
1. There is extensive discussion of currents flowing up and down dendrites starting on page 8, but it is only on page 11 that it is stated that this is referring to intracellular longitudinal current. It would be helpful for readers not familiar with the HNN modeling framework to state this explicitly on page 8, and perhaps to give a brief explanation of how these longitudinal intracellular currents provide predictions of the dipole sources generating the extracellular LFPs that are measured in MEG (cf. Fig. 1 of Neymotin et al. 2020 [14]).
Thank you. We’ve added a paragraph under “Biophysical model” in the Results (as well as a couple of sentences in the following paragraph) describing how the longitudinal currents give rise to MEG signals.

2. RSME values for the model fits to the data are given without units. I believe from Fig. 3 that the units should be nAm - these units should be given after all RSME values. Additionally, it might be beneficial to also state the RMSE values as a percentage of the amplitude (peak-to-peak, say) of the empirical waveforms, to help the reader appreciate the relative size of the error. Finally, the labels on panels A and B of Figs. 3, S1, and S5 should be "RMSE", rather than just "RMS".
We’ve added units and fixed the labels. Instead of expressing the RMSE values as a percentage we thought it would be more fitting to use R^2 values from linear regressions between the dipole waveforms from the data and those from the model. This gives a readily interpretable measure of fraction of variance explained by the model. We added this in the Methods section under “Model” as well as reported all the R^2 values.

In carrying out this new analysis, we also caught a mistake in the previous analysis: While recalculating the RMSE values, we realized that we were plotting the wrong dipoles and individual-layer responses in Figure 1 (what was being plotted were unoptimized model fits; what's plotted now is after optimization). We made this correction.
